# Depletion of microglia augments the dopaminergic neurotoxicity of MPTP

**DOI:** 10.1096/fj.201700833RR

**Published:** 2018-01-22

**Authors:** Xiaoxia Yang, Honglei Ren, Kristofer Wood, Minshu Li, Shenfeng Qiu, Fu-Dong Shi, Cungen Ma, Qiang Liu

**Affiliations:** * Department of Neurology, Tianjin Neurological Institute, Tianjin Medical University General Hospital, Tianjin, China;; † Department of Neurology, Barrow Neurological Institute, St. Joseph’s Hospital and Medical Center, Phoenix, Arizona, USA;; ‡ Department of Basic Medical Sciences, University of Arizona College of Medicine–Phoenix, Phoenix, Arizona, USA;; § Institute of Brain Science, Shanxi Datong University School of Medicine, Datong, China

**Keywords:** MPTP, Parkinson’s disease, neuroprotection

## Abstract

The activation of microglia and the various substances they produce have been linked to the pathologic development of Parkinson’s disease (PD), but the precise role of microglia in PD remains to be defined. The survival of microglia depends on colony-stimulating factor 1 receptor (CSF1R) signaling, and CSF1R inhibition results in rapid elimination of microglia in the central nervous system. Using a mouse PD model induced by 1-methyl-4-phenyl-1,2,3,6-tetrahydropyridine (MPTP) treatment, we showed that the depletion of microglia *via* the CSF1R inhibitor PLX3397 exacerbated the impairment of locomotor activities and the loss of dopaminergic neurons. Further, depletion of microglia augmented the production of inflammatory mediators and infiltration of leukocytes in the brain after MPTP exposure. Microglia depletion–induced aggravation of MPTP neurotoxicity was also seen in lymphocyte-deficient mice. In addition, the depletion of microglia did not affect the production of brain-derived neurotrophic factor, but it dramatically augmented the production of inflammatory mediators by astrocytes after MPTP treatment. Our findings suggest microglia play a protective role against MPTP-induced neuroinflammation and dopaminergic neurotoxicity.—Yang, X., Ren, H., Wood, K., Li, M., Qiu, S., Shi, F.-D., Ma, C., Liu, Q. Depletion of microglia augments the dopaminergic neurotoxicity of MPTP.

Parkinson’s disease (PD) is the second most common and debilitating neurodegenerative disorder, and is characterized by progressive loss of dopaminergic neurons and motor functions (1–3). Evidence indicates that neuroinflammation plays a crucial role in a cascade of pathologic events that lead to this loss of neurons and the progression of PD (2, 4, 5). As brain-resident immune cells, microglia are among the first responders to neural injury. Microglia engage in intimate cross talk with other intrinsic brain cells and leukocytes that infiltrate the brain from the periphery through the compromised blood–brain barrier (2, 4–7). Upon activation, microglia can produce such varied substances as proinflammatory cytokines, neurotoxic factors, and chemokines that promote inflammation and neurotoxicity, as well as anti-inflammatory and neurotrophic factors that restrict neuroinflammation and provide beneficial functions in neurodegenerative diseases (8–10). Thus, microglia are profoundly influential depending on the setting, timing, and types of diseases they encounter. However, the precise influence of microglia on brain inflammation and their contribution to dopaminergic degeneration remain poorly understood in the milieu of PD.

The survival of microglia, in contrast to that of macrophages in peripheral tissues, depends on signaling by colony-stimulating factor 1 receptor (CSF1R) (11–13). Because microglia are the major brain cells that express CSF1R, treatment with a CSF1R inhibitor such as PLX3397 results in a near-complete elimination of microglia from the brain with no apparent impact on other brain cells such as neurons, astrocytes, or oligodendrocytes (11, 12, 14, 15). Although PLX3397 was reported as a CSF1R inhibitor (12), it is also a potent multitargeted receptor tyrosine kinase inhibitor of CSF1R as well as c-Kit (mast/stem cell growth factor receptor) and FLT3 (FMS-like tyrosine kinase 3) (16, 17). As demonstrated, PLX3397 potently inhibits CSF1R and c-Kit over most other kinases (12, 16, 17). Moreover, once eliminated, microglia remain absent throughout the entire period of treatment, allowing for a well-delimited period in which the brain becomes a microglia-free environment (11, 12, 14, 15, 17, 18). Therefore, depletion of microglia using PLX3397 provides the opportunity to investigate the role of microglia in dopaminergic neurotoxicity of 1-methyl-4-phenyl-1,2,3,6- tetrahydropyridine (MPTP).

In this study, we have exploited this approach to deplete microglia and determine the influence of microglia on brain inflammation and dopaminergic neurotoxicity of MPTP. Our results demonstrate that the depletion of microglia exacerbates brain inflammation and dopaminergic neurotoxicity following MPTP treatment, thus revealing an uncircumscribed protective role of microglia in PD pathogenesis.

## MATERIALS AND METHODS

### Experimental animals

Male 7–8-wk-old C57BL/6L and Rag2^−/−^γc^−/−^ mice (Rag2/Il2rg compound mutant mice lacking T, B, NK, and NKT cells) were obtained from Charles River Laboratories (Wilmington, MA, USA) and Vital River Corp. (Beijing, China). This study was conducted in accordance with the U.S. National Institutes of Health guidelines for the use of laboratory animals. All experiments were approved by the institutional animal care and use committees of Tianjin Medical University General Hospital and Barrow Neurological Institute. All animals could freely access food and water, and were maintained in a temperature-controlled environment on a 12/12 h light–dark cycle. All animal experiments were designed, performed, and reported on according to the ARRIVE (Animal Research: Reporting of *In Vivo* Experiments) guidelines. Animals were randomly assigned to experimental groups.

### MPTP administration

C57BL/6 (B6) or Rag2^−/−^γc^−/−^ mice each received 4 injections of MPTP-HCl (20 mg/kg, i.p.; Sigma-Aldrich, St. Louis, MO, USA) in 2-h intervals. Seven days after the last injection, mice were euthanized as previously described (19–21). Littermate mice of the same sex were used as controls and were administered saline only.

### Compounds

PLX3397 (pexidartinib; Selleckchem, Houston, TX, USA) was dissolved in DMSO and the resultant solution was diluted with PBS. Mice were administered PLX3397 at a dosage of 40 mg/kg per day for 21 d prior to MPTP injection, which continued until these experiments ended, as previously described (12, 22).

### Motor function assessments

Systemic motor abilities of mice, specifically their coordination and balance, were assessed by rotarod testing as previously described (23, 24). Three trials, in which the rod’s rotational speed accelerated from 0 to 40 rpm, were performed on an automated accelerating rotarod apparatus (3 cm in diameter and 30 cm long, with a nonslip surface 20 cm above the base). Each trial lasted 15 min with a 30 min interval between trials. The results were expressed as the average time of 3 trials.

A pole test was used to determine the degree of bradykinesia. All mice were placed upright at the top of a rough-surfaced pole (1 cm in diameter and 50 cm in height) that was double-wrapped with gauze to prevent slipping. The time it took for each mouse to climb down to the floor was recorded, as previously described (25). In this study, the test was performed at d 7 after MPTP treatments with each experimental schedule.

### Immunostaining

As previously described (23, 26, 27), brains removed from test mice were frozen and later sectioned into 25-μm-thick slices before fixation with 4% paraformaldehyde for 30 min. To quantify tyrosine hydroxylase–positive (TH^+^) cells, brain sections were incubated with peroxidase sealant for 10 min, followed by 10% fetal bovine serum for 30 min. Thereafter, brain sections were incubated overnight with a primary antibody against TH [1:200 dilution (v/v); MAB318; Millipore, Billerica, MA, USA], followed by 1 h incubation at room temperature with a biotinylated secondary antibody (GK500710; Gene Tech, Shanghai, China). After staining with 3,3-diaminobenzidin [1:50 (v/v), GK500710; Gene Tech], sections were mounted in neutral balsam. Finally, the immunostained TH^+^ cells from the substantia nigra were counted in every 10th tissue section throughout each entire tissue block. In total, ∼8 sections per mouse brain were stained and there was an interval of ∼250 μm between any 2 adjacent brain sections. To count the brain-infiltrating immune cell subsets, brain tissue sections were incubated with anti-mouse CD4 [1:100 (v/v), sc-19641; Santa Cruz Biotechnology, Dallas, TX, USA], CD8 [1:100 (v/v), sc-7188; Santa Cruz Biotechnology], CD19 [1:100 (v/v), ab25232; Abcam, Cambridge, United Kingdom], CD335 [1:100 (v/v), sc-18161; Santa Cruz Biotechnology], CD169 [1:100 (v/v), MA1-80164; Thermo Fisher Scientific, Waltham, MA, USA], and Ly6G [1:50 (v/v), BP0075-1; Bio X Cell, West Lebanon, NH, USA] primary antibodies at 4°C overnight, and then incubated with Alexa Fluor 488–conjugated donkey anti-mouse, anti-rabbit, or anti-rat secondary antibodies [1:1000 (v/v); Thermo Fisher Scientific] at room temperature for 1 h. Nuclei were costained with DAPI (Abcam). Images were captured by fluorescence microscopy (Olympus BX-61; Tokyo, Japan).

### Flow cytometry

Quantitative analyses of immune cell subsets or cytokines prepared from brain tissues and stained with fluorochrome-conjugated antibodies followed, as previously described (26, 27). At d 7 post–MPTP administration, brains were harvested and homogenized using a 40 μm nylon cell strainer (Becton Dickinson, Franklin Lakes, NJ, USA) in PBS. Then 30% percoll (GE Healthcare Bio-Sciences, Uppsala, Sweden) was used to isolate cellular components from brain tissue, and the cells were stained with fluorochrome-conjugated antibodies. Antibodies were labeled with 1 of 5 fluorescent tags: FITC, phycoerythrin, perinidin chlorophyll, allophycocyanin, or phycoerythrin–cyanin 7. The following antibodies were used: CD3 (145-2C11; BD Biosciences, Franklin Lakes, NJ, USA), CD4 (RM4-5; BD Biosciences), CD8 (53–6.7; BD Biosciences), CD19 (1D3; BioLegend, San Diego, CA, USA), NK1.1 (natural killer 1.1; PK136; BD Biosciences), CD45 (30-F11; eBioscience, San Diego, CA, USA), CD11b (M1/70; eBioscience), F4/80 (EGF-like module-containing mucin-like hormone receptor-like 1; BM8; BioLegend), Ly6G (lymphocyte antigen 6 complex, locus G; 1A8; BioLegend), CD69 (H1.2F3; BioLegend), CD25 (PC61; BioLegend), and GLAST (glutamate aspartate transporter; NB100-1869; Novus Biologicals, Littleton, CO, USA). For intracellular staining, cells were fixed and permeabilized with a commercial solution (340973; BD Biosciences), and then stained with the following antibodies: IFN-γ (XMG1.2; BioLegend), TNF-α (MP6-XT22; BD Biosciences), IL-6 (MP5-20F3; eBioscience), iNOS (CXNFT; eBioscience), IP-10 (IFN-γ–induced protein 10; 6D4; Abcam), CCL2 (C-C motif chemokine ligand 2; 2H5; BioLegend), and CCL21 (59106; Novus Biologicals). Flow cytometric measurements were performed on a FACSAria (BD Biosciences) and analyzed using FACSDiva and FlowJo 7.6 software (FlowJo, Ashland, OR, USA).

### Real-time RT-PCR

Total RNA was extracted from substantia nigra and striata with Trizol reagent (Thermo Fisher Scientific), as previously described (26, 27). cDNA was transcribed using a TransScript First-Strand cDNA Synthesis SuperMix Kit (TransGen Biotech, Beijing, China) according to the manufacturer’s instructions. PCR was performed on a DNA Engine Opticon 2 real-time PCR detection system (Bio-Rad, Hercules, CA, USA) with corresponding primers (**Table 1**) and SYBR Green PCR Master Mix (Roche Diagnostics, Basel, Switzerland). The CT values for triplicate samples were averaged, and the data were analyzed with the ΔΔ*C_t_* method, where fold change = 2^−ΔΔ*Ct*^. Expression levels of mRNA were then reported as fold changes *vs.* control. Quantitative levels of mRNA were normalized to β-actin expression.

**TABLE 1.** Primer sequences for quantitative RT-PCR

Gene	Primer, 5′–3′	
	Forward	Reverse
*IL-1β*	GCTGCTTCCAAACCTTTGAC	AGCTTCTCCACAGCCACAAT
*TNF-α*	ACGGCATGGATCTCAAAGAC	GTGGGTGAGGAGCACGTAGT
*IL-2*	GAGCAGCTGTTGATGGACCT	TTTCAATTCTGTGGCCTGCT
*IL-4*	GCAACGAAGAACACCACAGA	TGCAGCTCCATGAGAACACT
*IL-6*	ACCGCTATGAAGTTCCTCTCTGCA	AAGCCTCCGACTTGTGAAGTGGT
*IL-8*	AAGGCTGGTCCATGCTCCT	CACAGACATCGTAGCTCTTGAGTG
*IL-10*	AAATAAGAGCAAGGCAGTGG	GTCCAGCAGACTCAATACACA
*IFN-γ*	ATCAGGCCATCAGCAACAA	ACCTGTGGGTTGTTGACCTC
*MIP-1α*	AGATTCCACGCCAATTCATC	CCCAGGTCTCTTTGGAGTCA
*iNOS*	GACGAGACGGATAGGCAGAG	CACATGCAAGGAAGGGAACT

### ELISA

Striatal and substantia nigra tissues (∼80 mg/mouse) were homogenized and supernatants were collected after centrifugation. Supernatants from brain homogenates were used to analyze the expression of pro–brain-derived neurotrophic factor (BDNF) and active BDNF by using commercial ELISA kits (Trust Specialty Zeal, San Francisco, CA, USA). The absorbance was measured with a microplate photometer (Thermo Fisher Scientific).

### Statistics

Sample size was determined by power analysis using a significance level of α = 0.05 with 80% power to detect significant differences. Power analysis and sample size calculations were performed with SAS 9.1 software (SAS Institute, Cary, NC, USA). All results were evaluated by investigators blinded to the treatment. Data are expressed as means ± sem. Statistical analyses were performed using GraphPad Prism software (La Jolla, CA, USA). The 2-tailed, unpaired Student’s *t* test was used to determine the significance of differences between 2 groups. One-way ANOVA, followed by a Tukey *post hoc* test were used for 3 or more groups. Two-way repeated ANOVA followed by Bonferroni posttests were performed for multiple comparisons. A value of *P* < 0.05 was considered significant.

## RESULTS

### PLX3397 treatment eliminates microglia in MPTP-treated mice

**Figure 1.**
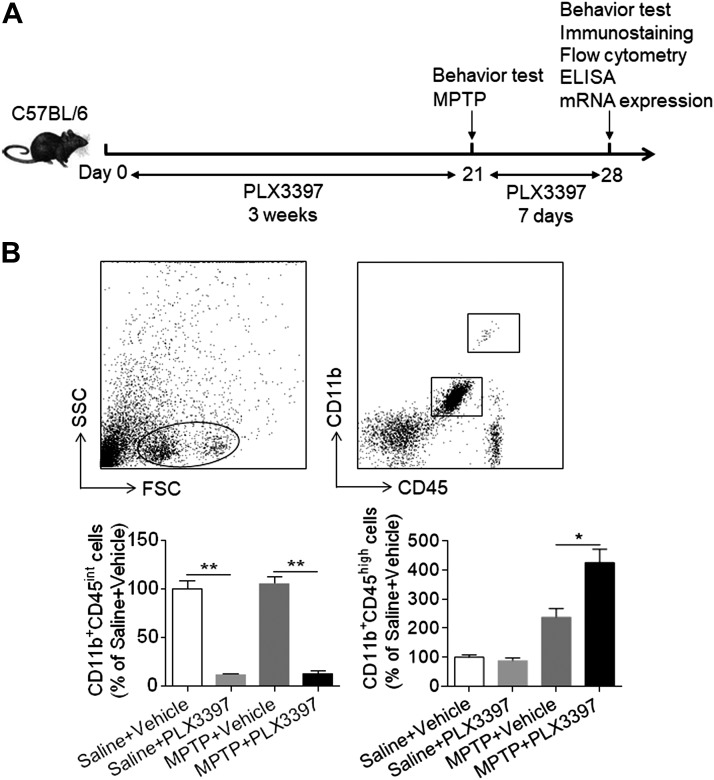
PLX3397 treatment eliminates microglia in the brain after MPTP exposure. *A*) Schematic shows the experimental design. C57BL/6 mice received PLX3397 or vehicle for 21 d prior to saline or MPTP administration (intraperitoneal injection). Mice continued to receive PLX3397 or vehicle until the experiments ended. At indicated time points after saline or MPTP administration, mice were subjected to behavioral tests (Figs. 2 and 4). Their isolated brain cells were used for immunostaining (Figs. 2–4) or flow cytometry (Figs. 1, 3, and 6), and their brain homogenates were used for ELISA (Fig. 5) or for measuring mRNA expression (Fig. 3). *B*) Flow cytometry plots illustrate the gating strategy for assessing brain microglia (CD11b^+^CD45^int^) and infiltrating macrophages (CD11b^+^CD45^high^). At d 7 after saline or MPTP injection, counts of microglia and infiltrating macrophages in C57BL/6 mice receiving PLX3397 or vehicle are shown as summarized. All data are presented as means ± sem; *n* = 10 mice/group. **P < 0.01.

C57BL/6 mice received PLX3397 or vehicle for 21 d prior to saline or MPTP treatment (**Fig. 1*A***). Thereafter, these mice continued to receive PLX3397 or vehicle until the end of experiment. At d 7 after saline or MPTP treatment, the efficacy of microglia elimination by PLX3397 was assessed using flow cytometry. PLX3397 administration resulted in ∼90% reduction of microglia (CD11b^+^CD45^int^) in MPTP-treated mice but did not significantly affect the number of macrophages (CD11b^+^CD45^high^) (Fig. 1*B*). In addition, the efficacy of microglial depletion in MPTP-treated mice was similar to that in saline-treated mice (Fig. 1*B*). These data demonstrate that PLX3397 treatment can effectively eliminate microglia following MPTP treatment.

### Depletion of microglia augments neurodeficits and the loss of dopaminergic neurons in mice subjected to MPTP treatment

**Figure 2.**
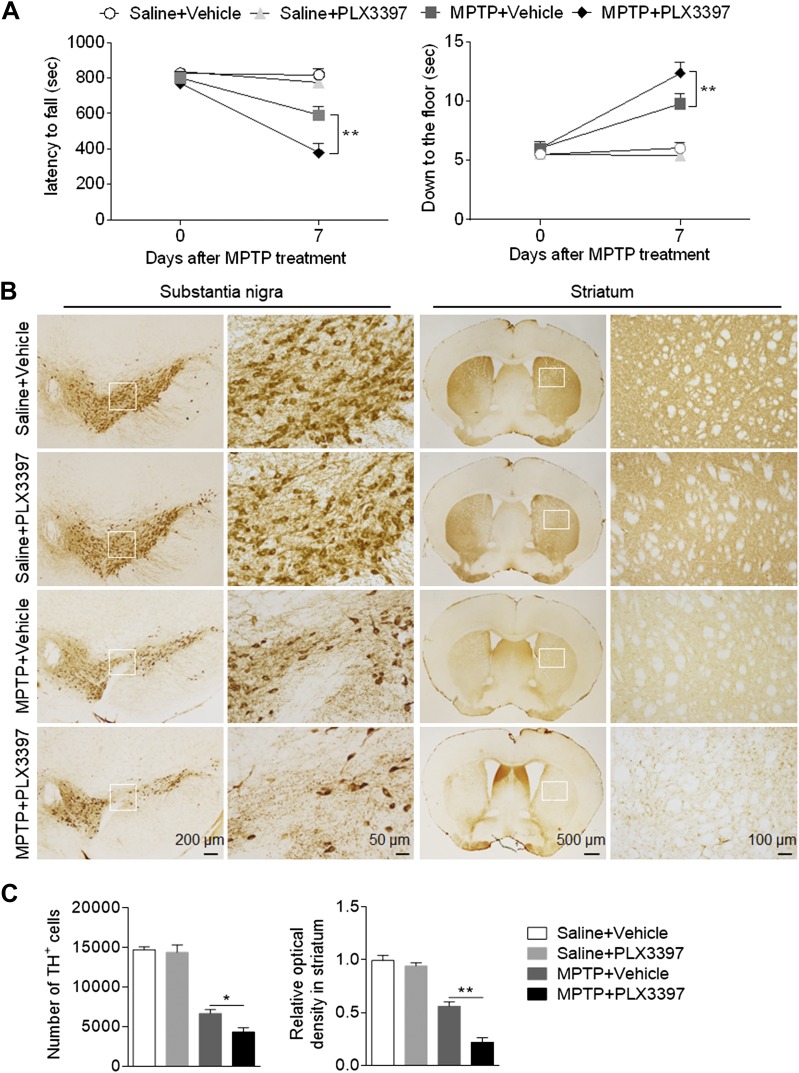
Depletion of microglia exacerbates nigrostriatal dopaminergic degeneration following MPTP treatment. C57BL/6 mice received PLX3397 or vehicle for 21 d prior to saline or MPTP administration (intraperitoneal injection). Mice continued to receive PLX3397 or vehicle until the experiments ended. *A*) Effects of microglial depletion on motor skills following MPTP treatment in C57BL/6 mice. Motor function was assessed by measuring the latency to fall and the ability to climb down to the floor at indicated time points following saline or MPTP treatment. *B*) Immunostaining with TH shows TH^+^ cells in substantia nigra and the immunoreactivity of TH in striata of groups of C57BL/6 mice receiving PLX3397 or vehicle at d 7 following saline or MPTP treatment. *C*) Bar graphs show the effects of microglial depletion on the number of TH^+^ cells in substantia nigra and the immunoreactivity of TH in striata at d 7 following saline or MPTP treatment. All data are presented as means ± sem; *n* = 10 mice/group. *P < 0.05, **P < 0.01.

To determine whether microglial depletion affects dopaminergic neurotoxicity following MPTP treatment, we assessed the motor function, number of TH^+^ cells in substantia nigra, and optical density of TH immunoreactivity in striata of mice treated with either PLX3397 and saline, PLX3397 and MPTP, or vehicle and saline as well as those treated with vehicle and MPTP. At d 0 and 7 after saline or MPTP treatment, motor function was assessed by measuring the time between latency and falling in the rotarod test and the time required to reach the floor in the pole test. Microglial depletion aggravated MPTP-induced motor deficits (**Fig. 2*A***). In contrast, microglial depletion did not alter motor function in mice receiving saline treatment (Fig. 2*A*), suggesting that a loss of microglia exacerbates MPTP-induced motor deficits.

Next, we measured the counts of TH^+^ cells in substantia nigra and the optical density of TH immunoreactivity in striata at d 7 after saline or MPTP treatment. We found that the counts of TH^+^ cells in substantia nigra were significantly reduced in MPTP-treated mice subjected to microglial depletion (Fig. 2*B, C*). Similarly, microglial depletion reduced the immunoreactivity of TH in striata of mice receiving MPTP treatment, but not in mice receiving saline treatment (Fig. 2*B, C*). Together, these results suggest that microglial depletion augments MPTP-induced dopaminergic neurotoxicity.

### Depletion of microglia promotes leukocyte infiltration and local inflammation

**Figure 3.**
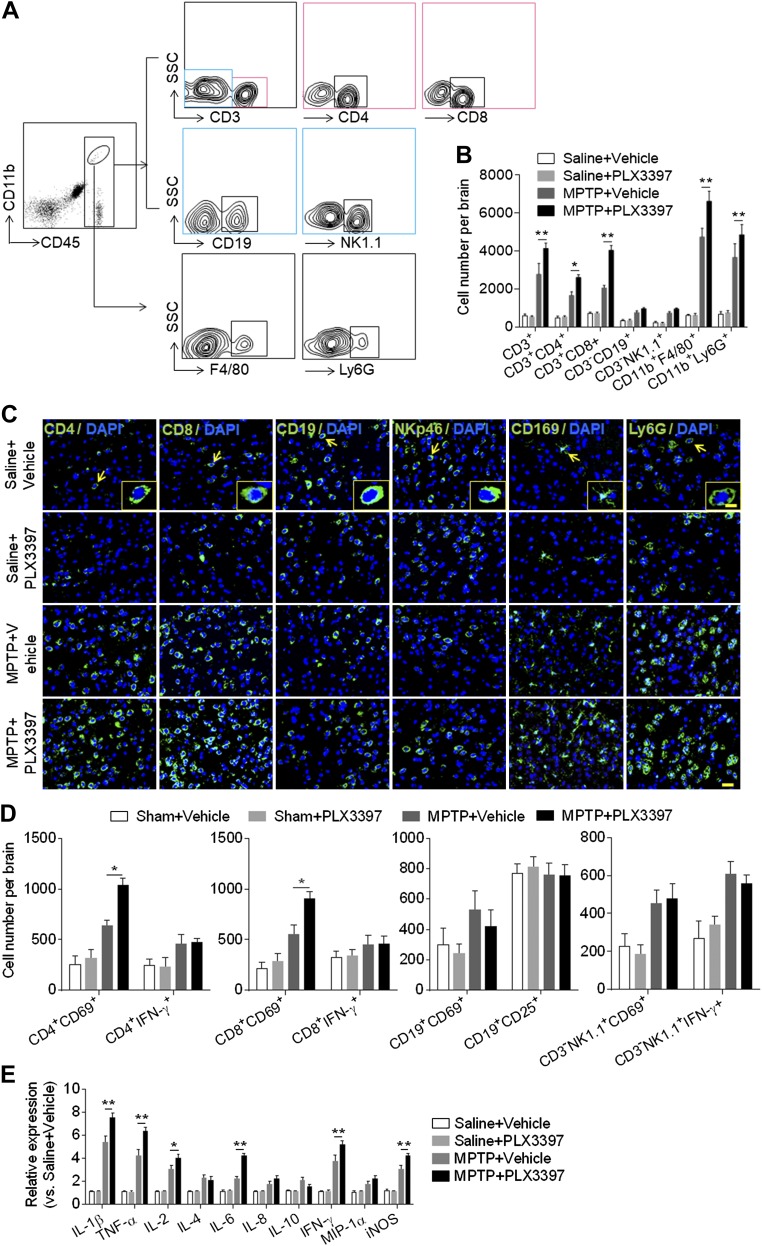
Depletion of microglia augments leukocyte infiltration and brain inflammation following MPTP treatment. C57BL/6 mice received PLX3397 or vehicle for 21 d prior to saline or MPTP administration (intraperitoneal injection). Mice continued to receive PLX3397 or vehicle until the experiments ended. At d 7 after saline or MPTP treatment, single cell suspensions were prepared from substantia nigra and striatal tissues. *A*) Flow cytometry plots show the gating strategy of infiltrating leukocyte subsets including T cells (CD45^high^CD3^+^), CD4^+^ T cells (CD45^high^CD3^+^CD4^+^), CD8^+^ T cells (CD45^high^CD3^+^CD8^+^), B cells (CD45^high^CD3^−^CD19^+^), NK cells (CD45^high^CD3^−^NK1.1^+^), monocytes and macrophages (CD45^high^CD11b^+^F4/80^+^), and neutrophils (CD45^high^CD11b^+^Ly6G^+^). *B*) Quantification of infiltrating lymphocytes, macrophages, and neutrophils from groups of mice receiving indicated treatments. *C*) Immunostaining of CD4^+^ T cells (CD4, green), CD8^+^ T cells (CD8, green), B cells (CD19, green), NK cells (NKp46, green), monocytes and macrophages (CD169, green), neutrophils (Ly6G, green), and DAPI (blue). *D*) Summarized results show the expression of CD69, IFN-γ, and CD25 in infiltrating lymphocyte subsets from groups of mice receiving indicated treatment. Scale bars, 50 µm; 10 µm (inset). *E*) Bar graphs show the mRNA expression of IL-1β, TNF-α, IL-2, IL-4, IL-6, IL-8, IL-10, IFN-γ, MIP-1α (macrophage inflammatory protein-1α), and iNOS in substantia nigra and striatal tissues from groups of mice receiving the indicated treatment. All data are presented as means ± sem; *n* = 9 mice/group. *P < 0.05, **P < 0.01.

Leukocytes infiltrating the brain are prominent contributors to local inflammation and dopaminergic neuron death after MPTP exposure (21, 28–30). Therefore, we aimed to determine the effects of microglial elimination on leukocyte infiltration following MPTP exposure. Using flow cytometry, we quantified the counts of infiltrating leukocytes in groups of mice receiving PLX3397 and saline, PLX3397 and MPTP, vehicle and saline, or vehicle and MPTP. At d 7 after MPTP treatment, we found that microglial depletion augmented the infiltration of CD4^+^ T cells (CD45^high^CD3^+^CD4^+^), CD8^+^ T cells (CD45^high^CD3^+^CD8^+^), monocytes and macrophages (CD45^high^CD11b^+^F4/80^+^), and neutrophils (CD45^high^CD11b^+^Ly6G^+^) (**Fig. 3*A, B***), suggesting that microglia may restrict MPTP-induced leukocyte infiltration. Results from immunostaining of infiltrating leukocytes in brain sections were consistent with the findings of flow cytometry analysis (Fig. 3*C*). In addition, we found that microglial depletion resulted in a significant increase of activation marker CD69 in CD4^+^ T and CD8^+^ T cells after MPTP treatment (Fig. 3*D*). To further determine the impact of microglial depletion on MPTP-induced local inflammation, we measured the expression of cytokines and chemokines in the substantia nigra and striatal tissues. PLX3397 treatment significantly up-regulated the expression of proinflammatory cytokines including IL-1β, TNF-α, IL-2, IL-6, IFN-γ, and iNOS (Fig. 3*E*), suggesting that microglial depletion augments MPTP-induced local inflammation.

### Microglial depletion–enhanced dopaminergic neurotoxicity of MPTP does not entirely depend on lymphocytes

**Figure 4.**
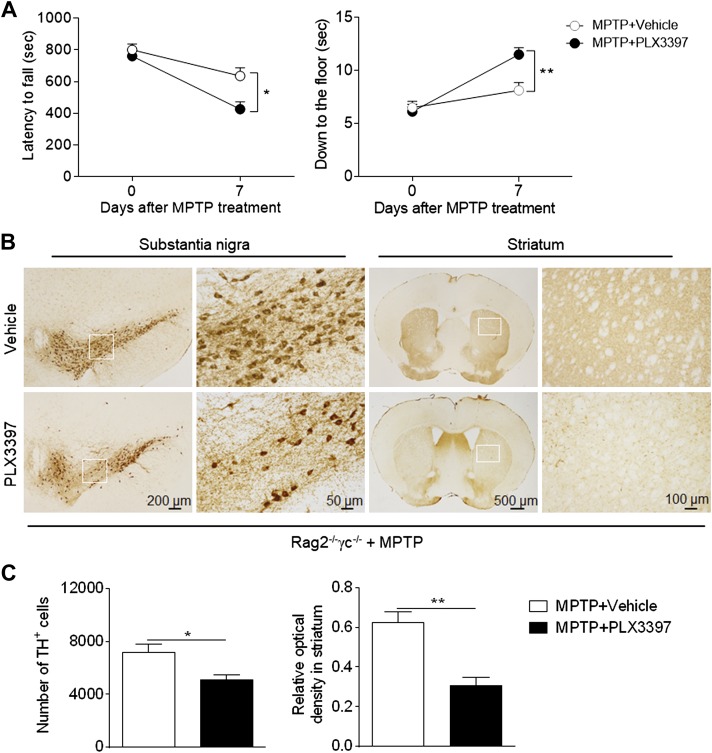
A decrease in microglia-induced exacerbation of PD severity does not depend entirely on lymphocytes. Rag2^−/−^γc^−/−^ mice received PLX3397 or vehicle for 21 d prior to saline or MPTP administration (intraperitoneal injection). Mice continued to receive PLX3397 or vehicle until the experiments ended. *A*) Effects of microglial depletion on motor skills following MPTP treatment in Rag2^−/−^γc^−/−^ mice. Motor function was assessed by measuring the latency to fall and the ability to climb down to the floor at indicated time points following saline or MPTP treatment. *B*) Immunostaining with TH shows TH^+^ cells in substantia nigra and the immunoreactivity of TH in striata of groups of Rag2^−/−^γc^−/−^ mice receiving PLX3397 or vehicle at d 7 following saline or MPTP treatment. *C*) Bar graphs show the effects of microglial depletion on the number of TH^+^ cells in substantia nigra and the immunoreactivity of TH in striata at d 7 following saline or MPTP treatment. Bar graphs illustrate the latency to fall and the ability to climb down to the floor of Rag2^−/−^γc^−/−^ mice receiving PLX3397 or vehicle at d 7 after MPTP injection. All data are presented as means ± sem; *n* = 9 mice/group. *P < 0.05, **P < 0.01.

Because lymphocytes are considered to be a key contributor to brain inflammation and dopamine neuron death after MPTP exposure (21, 28–30), we sought to understand whether microglia depletion–related augmentation of MPTP’s dopaminergic neurotoxicity requires lymphocytes. For this purpose, we assessed the impact of microglial depletion on the dopaminergic neurotoxicity of MPTP in lymphocyte-deficient mice (Rag2^−/−^γc^−/−^mice lacking T, B, NK, and NKT cells). Microglial depletion still aggravated MPTP-induced motor deficits and dopaminergic degeneration in the substantia nigra and striata (**Fig. 4*A, C***), indicating that microglia depletion–induced augmentation of the dopaminergic neurotoxicity of MPTP does not entirely depend on lymphocytes.

### BDNF expression is not significantly altered by microglial depletion in mice subjected to MPTP treatment

**Figure 5.**
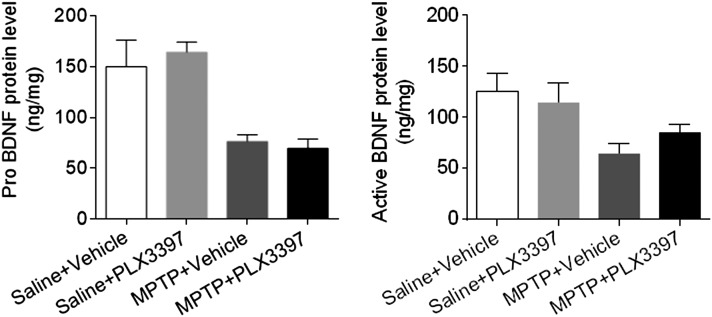
Microglial depletion does not alter BDNF expression in the brain after MPTP treatment. C57BL/6 mice received PLX3397 or vehicle for 21 d prior to saline or MPTP administration (intraperitoneal injection). Mice continued to receive PLX3397 or vehicle until the experiments ended. At d 7 after saline or MPTP injection, tissue homogenates were prepared from substantia nigra and striata. Bar graphs show the effect of microglial depletion on the protein levels of pro- and active BDNF in groups of mice receiving the indicated treatments. All data are presented as means ± sem; *n* = 8 mice/group.

The activation of microglia triggers a release or production of BDNF, which could help to protect dopaminergic neurons from damage caused by MPTP (31, 32). To test this possibility, we examined the protein levels of the two forms of BDNF, pro-BDNF and active BDNF, in substantia nigra and striatal tissues obtained from groups of mice administered PLX3397 and saline, PLX3397 and MPTP, vehicle and saline, or vehicle and MPTP. At d 7 after MPTP treatment, we found that microglial depletion did not alter pro-BDNF expression in substantia nigra and striatal tissues, but there was a slight increase in active BDNF expression (**Fig. 5**).

### Microglial depletion augments the MPTP-induced astrocyte responses

**Figure 6.**
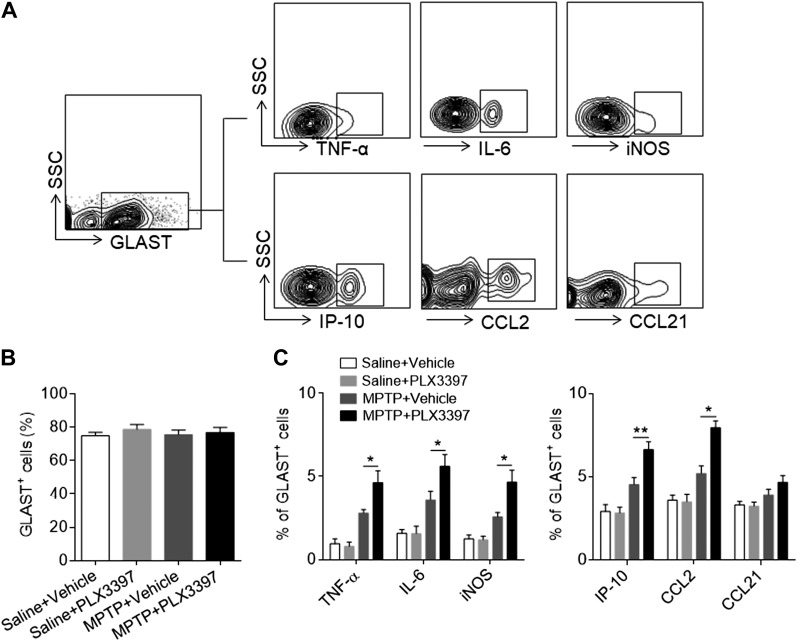
Depletion of microglia promotes astrocyte responses following MPTP treatment. C57BL/6 mice received PLX3397 or vehicle for 21 d prior to saline or MPTP administration (intraperitoneal injection). Mice continued to receive PLX3397 or vehicle until the experiments ended. At d 7 after saline or MPTP injection, single cell suspensions were prepared from substantia nigra and striatal tissues. *A*) Flow cytometry plots show the gating strategy for astrocytes (GLAST^+^) and their expression of TNF-α, IL-6, iNOS, IP-10, CCL2, and CCL21. *B*, *C*) Bar graphs show the effects of microglial depletion on the expression of TNF-α, IL-6, iNOS, IP-10, CCL2, and CCL21 in astrocytes at d 7 after MPTP treatment. All data are presented as means ± sem; *n* = 9 mice/group. *P < 0.05, **P < 0.01.

Astrocytes are the most abundant cell type in the brain and possess a potent proinflammatory function after brain injury (33–35). We therefore measured the astrocyte response after saline or MPTP treatment in recipients of either PLX3397 or vehicle. Although PLX3397 treatment did not significantly affect the number of astrocytes in mice receiving either saline or MPTP (**Fig. 6*A, B***), MPTP treatment resulted in a significant increase in astrocytes that expressed proinflammatory factors including TNF-α, IL-6, and iNOS, and chemokines including IP-10 and CCL2 (Fig. 6*C*). PLX3397 treatment led to a dramatic up-regulation of TNF-α, IL-6, and iNOS as well as IP-10 and CCL2 in astrocytes after MPTP treatment (Fig. 6*C*). By contrast, PLX3397 treatment did not affect the astrocyte response in saline-treated mice (Fig. 6*C*). Together, these results demonstrate a significantly increased astrocyte response after microglial depletion, suggesting that this enhanced proinflammatory response of astrocytes may be involved in microglia depletion–induced aggravation of MPTP neurotoxicity.

## DISCUSSION

This study provides novel evidence that microglia engage in protective action against MPTP-induced dopaminergic neurotoxicity. As documented here, microglial depletion enhances the infiltration of leukocytes into the brain and causes local inflammation in the substantia nigra and striata after MPTP treatment. Although the microglia depletion–induced augmentation of MPTP neurotoxicity does not depend entirely on lymphocytes or relate to BDNF production, it is noteworthy that microglial depletion significantly enhances an MPTP-induced astrocyte response that may promote the neuroprotective effects of microglia. In support of a beneficial role of microglia against the dopaminergic neurotoxicity of MPTP, our findings also suggest that microglia are pivotal to neural-immune crosstalk in neurodegenerative diseases.

Considerable evidence points to microglia-mediated neuroinflammation as a landmark of PD (9, 10, 36). After activation, microglia can produce a range of reactive oxygen species, including NO and superoxide anion, and release proinflammatory cytokines that exacerbate dopaminergic degeneration and neurodeficits in PD (4, 8–10). We observed that, under certain circumstances, microglia also enhance neuron survival by releasing trophic and anti-inflammatory factors (37, 38). Microglia-derived neurotrophic factor reportedly promotes neuron survival and rescues injured dopaminergic neurons in animal models and in a clinical trial of PD (37, 39–41). No direct proof has been found to confirm whether microglia-mediated neuroinflammation is the cause or the consequence of dopaminergic neuron degeneration, however; therefore, we are unable to explain how microglial activation produces detrimental or beneficial effects in PD. Nevertheless, in line with previous findings, we show here that microglial depletion exacerbates the dopaminergic neurotoxicity of MPTP. Together with the data showing augmented neuroinflammation after microglial depletion in MPTP-treated animals, these results allow us to infer a protective role for microglia in PD.

To determine the underlying mechanisms by which microglia protect against dopaminergic neurotoxicity of MPTP, we examined immune responses in the brain. Other than the production of cytokines and chemokines, microglia have been reported to interact intimately with infiltrating lymphocytes that can worsen MPTP-induced dopaminergic degeneration (21, 28–30). In support of this view, we found that the depletion of microglia led to enhanced infiltration of lymphocytes and heightened levels of proinflammatory factors in the brain. However, the protective role of microglia against MPTP was still seen in lymphocyte-deficient mice. Therefore, we speculate that lymphocytes may not be the only factor involved in worsening MPTP neurotoxicity after microglial depletion. Microglia-derived trophic factors have also been demonstrated to enhance neuron survival and attenuate the death of dopaminergic neurons in PD (37, 40, 41). Although our finding shows that microglial depletion does not affect the level of BDNF in MPTP-treated animals, we cannot exclude the possibility that microglia may produce beneficial trophic factors or anti-inflammatory cytokines to modulate neuroinflammation. In addition, because lymphocytes are elevated in the absence of microglia after MPTP exposure, it would be interesting to test whether microglia act as suppressive myeloid antigen–presenting cells in this setting. Nevertheless, the precise operating mechanisms though which microglia confer protection in PD require further investigation.

Astrocytes are the most abundant type of brain cells that acquire the capacity to produce proinflammatory factors and to present antigen after brain injury, properties that impact neuron survival and function (4, 5, 34). This suggests that the activation of astrocytes may promote dopaminergic degeneration in PD. Indeed, evidence shows that an enhanced astrocyte response contributes to the death of dopaminergic neurons in the substantia nigra (33, 42). In addition, we found that MPTP-induced astrocyte production of proinflammatory factors increased significantly after microglial depletion. Therefore, we hypothesize that microglia can provide neuroprotective effects by inhibiting MPTP-induced astrocyte responses. This possibility requires further investigation; however, the possible multifaceted functions of glial cells cannot be excluded from study.

Although PLX3397 was utilized as a CSF1R inhibitor (12), it is also a multitargeted receptor tyrosine kinase inhibitor of CSF1R, c-Kit, and Flt3 (16, 17). Reportedly, PLX3397 inhibits CSF1R and c-Kit over most other kinases (12, 16, 17). In addition to microglia, other myeloid cells such as monocytes and macrophages also express CSF1R (11–13), suggesting that PLX3397 treatment may also affect peripheral immune responses that may contribute to the protective effect of PLX3397 treatment in MPTP-treated animals. However, in our and others’ previous studies, the depletion of microglia using PLX3397 had no discernable impact on neurologic function or baseline brain inflammation status under physiologic conditions (12, 14, 17). Those previous studies support our theory that the beneficial effect of PLX3397 in quelling neuroinflammation and MPTP neurotoxicity is caused by the removal of microglia. That said, we cannot conclude that the protective effect observed by using PLX3397 in this study resulted entirely from selective CSF1R inhibition, because some off-target effects may have participated. In future studies, we will use more selective CSF1R inhibitors to accurately measure the benefit of selective CSF1R inhibition in animal models of PD. In summary, our data suggest that microglia orchestrate neuroinflammation and protect against the dopaminergic neurotoxicity of MPTP.

## Abbreviations

BDNF: brain-derived neurotrophic factor
CSF1R: colony-stimulating factor 1 receptor
MPTP: 1-methyl-4-phenyl-1,2,3,6-tetrahydropyridine
PD: Parkinson’s disease
TH: tyrosine hydroxylase
